# Design and Efficacy of a Speaker Verification Method Combining CNN and Transformer for Secure Access Control

**DOI:** 10.3390/s26165101

**Published:** 2026-08-12

**Authors:** Xiao Li, Xiao Hu, Kun Niu, Ling Tuo

**Affiliations:** 1 School of Computer Science, Beijing University of Posts and Telecommunications, Beijing 100876, China; lixiao1911@bupt.edu.cn; 2 Academy of Broadcasting Science, National Radio and Television Administration, Beijing 100866, China; tuoling@abs.ac.cn

**Keywords:** speaker verification, CNN, transformer

## Abstract

In the rapidly evolving landscape of computer and mobile applications, the demand for secure access control has become increasingly pivotal. This paper introduces a speaker verification method aimed at remotely verifying an individual’s claimed identity, so as to achieve access authorization. The primary objective is to develop a deep learning network capable of eliminating redundant and irrelevant information while learning robust deep speaker embedding descriptors that capture speaker-specific idiosyncrasies. This paper presents an AI framework combining convolutional neural network and transformer architectures, enhanced by (1) a stereoscopic attention mechanism that computes fine-grained attention weights across frequency, time, and channel dimensions with fewer parameters than existing CBAM or SE modules, and (2) a multi-feature aggregation mechanism that fuses supervised and unsupervised features at the utterance level to maximize complementary speaker information. These innovations introduce a new perspective for integrating acoustic and articulatory features, effectively addressing the challenges related to short-segment speech and cross-domain scenarios. Experimental results demonstrate that the AI framework achieves competitive performance under domain mismatch conditions. The methodology acts as a catalyst for advancing application authorization, highlighting the transformative potential of AI-driven innovations in the software engineering field.

## 1. Introduction

With the advent and ubiquity of the Internet, coupled with the exponential advancement of computer software and mobile applications, access control has become a crucial aspect of human–computer interaction. Speaker verification (SV) is a widely adopted access control method for human–computer interaction. It is an interdisciplinary domain bridging linguistics, technology, and security [1]. It refers to the process of verifying an individual’s claimed identity automatically based on unique vocal patterns, which convey valuable information about identity, culture, and even emotional state [2,3]. SV can be categorized into text-dependent SV (TD-SV) and text-independent SV (TI-SV) [4]. TD-SV requires users to utter specific phrases or keywords. For example, Xiaomi smart devices allow a fixed Mandarin Chinese phrase such as “Xiao-ai-tong-xue” for wake-up commands [5]. In contrast, TI-SV allows users the flexibility to speak freely, accommodating a wide range of phonetic content, speaking styles, and environmental conditions. This increased variability contributes to the widespread adoption of TI-SV, as it enhances the system’s robustness and adaptability in various real-world scenarios, from forensic investigation [3,6] to device control and secure access [7]. For example, TI-SV enhances device control and secure access, enabling users to operate mobile applications or smart devices through voice commands, instead of manually interacting with screens or pressing buttons after entering passwords. TI-SV plays a pivotal role in biometric authentication systems, offering a non-intrusive method for access control through voiceprint verification.

The development of TI-SV technology is confronted with significant challenges, among which the length of speech segments, the variability of accents, background noise, and the complexity of human language are the most prominent issues [8,9]. A series of efforts have been made to explore solutions or alleviate these challenges in recent years. Previous TI-SV methods have largely relied on Gaussian Mixture Model-Universal Background Model (GMM-UBM) based i-vector systems [10,11]. However, these i-vector-based approaches exhibit vulnerability to lexical diversity, particularly when dealing with short spoken segments [12,13]. This limitation arises because these models may struggle to capture sufficient speaker-specific information from brief or less varied speech segments. In the past, the Gaussian Mixture Model (GMM), which demonstrates that each speaker’s features align with a Gaussian distribution, was employed in the earliest TI-SV models. In [11], GMM-UBM serves as an alternative approach for speaker representation, requiring a substantial amount of training data. To address the differences between speakers and channels, the GMM super vector [14] is introduced, stacking the means of all GMM components in a high-dimensional space. Subsequently, the GMM-UBM-based method [15] is proposed, mapping the super vector to a low-dimensional numerical vector. However, the computational cost of the GMM super vector is high [16]. These approaches face challenges in encoding overlooked information in the channel dimension, resulting in elevated dependence on the length of speech segments.

In recent years, the TI-SV task has evolved towards AI-based approaches [16]. The significant advancements in deep neural networks (DNNs) [17,18,19] and the growing availability of extensive speech datasets [20,21,22] have prompted a transition from traditional methodologies to more sophisticated artificial intelligence (AI) solutions in recent research. These newer approaches predominantly aim to learn deep speaker embedding (DSE) from speech segments. By leveraging the powerful feature extraction capabilities of DNNs, these AI methods can capture richer and more discriminative speaker characteristics, thereby improving the robustness and accuracy of TI-SV systems, especially in handling short and variable speech inputs. These paradigms can be classified into frame-level and utterance-level approaches. Frame-level systems employ DNNs to derive DSE from individual frames, thereby embodying speaker information. The d-vector [23] is a prototypical instance, which integrates fully connected (FC) layers with maxout activations, and it successfully introduces DNNs for DSE learning into the TI-SV field. In contrast, the utterance-level systems leverage DNNs to extract DSE from entire spoken segments, incorporating various pooling techniques such as temporal average pooling [20,24], statistical pooling [25], and dictionary-based methods [26,27], to fuse frame-level features into a unified DSE descriptor. By aggregating information across a series of frames, utterance-level systems can capture more comprehensive speaker-related characteristics, enhancing robustness against accent changes and background noise. Notable utterance-level systems include x-vector [25] and r-vector [28], which have demonstrated superior performance compared to frame-level systems. In [25], a TDNN-based x-vector with a statistical pooling layer is proposed to project variable-length utterances into a fixed-length DSE. Notably, the operation of TDNN is conceptually equivalent to one-dimensional (1D) convolutional neural network (CNN), producing intermediate layer outputs with a 2D tensor shape. In [28], ResNet-based r-vector also achieves an impressive performance boost. ResNet is a type of 2D CNN, which regards the input signal as single-channel 2D gray-level images and extracts 3D feature maps. The significant successes of x-vector and r-vector have proven the efficacy of applying CNNs to TI-SV. Xie et al. [29] propose a thin ResNet encoder and a ghost VLAD-based aggregation to encode the spectrogram input and fuse frame-level features into utterance-level embedding. By evaluating the network architecture, it can be seen that the approach is significantly inspired by r-vector. Hong et al. [30] propose an improved TDNN architecture, which integrates TDNN with statistics pooling at each layer for considering the variation in temporal context. The approach is a variant of x-vector, which benefits speaker characteristic capturing. Choi et al. [31] propose a Temporal-Bottleneck ResNet, which integrates a transposed convolution that enriches the temporal information. The approach utilizes the nature of statistics pooling by capturing and retaining frame-level contexts through a unique temporal bottleneck configuration in its building blocks, and shows a substantially increased performance compared to raw ResNet. However, these approaches only extract DSE from spectrogram-based acoustic features, without considering the amplitudes of the raw speech signals.

Regarding utterance-level systems, both average pooling and statistical pooling treat all frames equally by assigning uniform weights to each frame’s features [29,32]. To generate more discriminative utterance-level DSE, attention mechanisms [33,34,35,36,37] have recently gained significant attention for their ability to explore speaker-specific information in speech signals. Utterance-level approaches [36,38] that integrate DNNs with attention mechanisms have demonstrated notable improvements in TI-SV performance. Many studies [33,34,37] introduce attention mechanisms for frame-level feature aggregation, emphasizing more discriminative frames. Many other studies seek to integrate attention mechanisms into the front-end, inherently enhancing the representation capabilities of TI-SV models. The Convolutional Block Attention Module (CBAM) [35] and the Squeeze-and-Excitation (SE) module [36] are two front-end attention mechanisms. These modules generate channel-specific attention weights to refine feature representations within DNNs, enhancing DSE learning beyond merely emphasizing certain frames. Attention mechanisms have markedly improved TI-SV performance by emphasizing essential local features and suppressing irrelevant ones. Recent TI-SV approaches that incorporate CNNs with attention mechanisms have proven beneficial for exploring speaker-related feature regions in input signals. Refs. [29,33,37] utilize self-attention for frame-level feature aggregation. Ref. [39] proposes attentive-statistics pooling by combining an attention mechanism with statistics pooling for frame-level feature aggregation. Refs. [40,41] introduce the idea of multi-head attention for frame-level feature aggregation, outperforming baselines by a significant margin. These approaches employ attention techniques only on the features generated by the front-end and not throughout the entire front-end model. As opposed to these approaches, many researchers endeavor to incorporate attention mechanisms throughout the front-end pipeline, naturally boosting the feature representation abilities of TI-SV models. The Convolutional Block Attention Module (CBAM) [35] and the Squeeze-and-Excitation (SE) attention module [36] are two well-known attention mechanisms throughout the front-end. CBAM uses channel attention and spatial attention, exploiting inter-channel and inter-spatial relationships of features. SE attention module uses channel attention to determine the attention weights for different channel positions. These two modules do not leverage frequency-specific or time-specific attention information, as features from different frequency bands or time regions within the same channel are assigned the same weight. In order to tackle the problem, a series of works, e.g., frequency-wise SE (fw-SE) [42], multi-scale frequency-channel attention (MFA) [43] and f-CBAM [35], are proposed for capturing additional dimension-specific information. However, these modules fail to leverage time-wise, frequency-wise and channel-wise information simultaneously. Despite the effectiveness of these attention modules, their inclusion significantly increases the model size. Recent studies have further explored lightweight architectures and efficient training strategies for speaker verification. For instance, a broadcasting CNN-Transformer architecture with knowledge distillation has been proposed to reduce model parameters while maintaining competitive performance [44]. Meanwhile, Transformers have recently demonstrated significant potential across a range of audio detection tasks, including sound event detection and speech recognition, as reviewed in [45].

To address the challenges of short-duration speech and cross-domain conditions, in this paper, the cornerstone lies in the employment of self-attention network supervised learning and Wav2Vec2 [38] based unsupervised learning to discern unique vocal patterns with increased precision, constructing a unified DSE learning framework with a shared multi-feature integration mechanism. Speaker-specific evidence is captured from two types of features in a speech segment. Such a multi-feature integration mechanism facilitates information sharing among supervised features and unsupervised features at the utterance level and hence maximizes the speaker information. Our contributions can be summarized as follows:

- **Proposal of Methodological**. Our work proposes several key elements that distinguish it from existing methodologies. Firstly, unlike conventional attention mechanisms that operate on only one or two dimensions, we propose a lightweight stereoscopic attention module that calculates fine-grained attention weights across three dimensions—frequency, time, and channel—simultaneously. This 3D attention design captures richer speaker-specific patterns while using fewer learnable parameters than existing 2D attention modules. Moreover, a proposed multi-feature aggregation mechanism enables the utterance-level embedding descriptors to be embedded not only in supervised features but also in unsupervised features. By fusing supervised (spectrogram-based) and unsupervised (raw waveform-based) features at the utterance level, our framework maximizes complementary speaker information—a strategy that, to our knowledge, has not been previously explored in this specific combination. Additionally, our approach effectively mitigates the short speech problem by leveraging 2 s segments from the VoxCeleb dataset. It represents a significant advancement over existing methods, which often depend on longer audio samples for reliable performance.
- **Network Structure Improvements**. Our work enhances several renowned neural network architectures for adapting to the TI-SV task. On the one hand, innovative adjustments are made to the original 50-layer residual network (ResNet-50) for extracting DSE from spectrogram-based acoustic features. Compared to raw ResNet-50, the modified ResNet-50 has fewer learnable parameters. On the other hand, the unsupervised and pre-trained Wav2Vec2 framework is strengthened with dense layers and an attention statistics pooling layer, discerning unique vocal patterns from the raw speech signal without spectrogram-based sampling.
- **Fusion of DNNs**. A stereoscopic attention residual network (**SARN**), a Wav2Vec2-based TDNN (**W2V2-TDNN**) and a shared multi-feature aggregator are proposed, respectively. By merging these techniques, a hybrid approach is offered for elevating the performance of TI-SV. Experimental results show that the synthesis alleviates the short speech problem and enhances the robustness against cross-domain issues. It benefits speaker-related information learning, and experimental results verify the superiority of our hybrid approach.

The remainder of this paper is organized as follows. Section 2 details the proposed methodology. Section 3 describes the experimental setup. Section 4 presents and discusses the experimental results. Section 5 concludes the paper. Finally, the author contributions, funding sources, data availability statement, acknowledgments, and conflicts of interest are stated.

## 2. Methodology

### 2.1. CNN Approach

This paper presents a novel 50-layer residual network (ResNet-50) variant for TI-SV, termed **S**tereoscopic **A**ttention **R**es**N**et (**SARN**). The architecture incorporates a plug-and-play Stereoscopic Attention Module (SAM) while applying channel-size reduction to the ResNet-50. Compared to raw ResNet-50, the modifications significantly reduce the number of learnable parameters without compromising performance.

#### 2.1.1. Stereoscopic Attention Module

Channel-wise attention mechanisms like CBAM and SE rely on MLP-based implementations, resulting in substantial parameter counts, quantified as $2Cd+2k^{2}$ and 2Cd [2], respectively, where C represents the input channel size, d denotes the hyper-parameter of the MLP, and k signifies the convolution kernel size. As the network depth increases, C grows significantly, further amplifying the parameter burden. To address this, we propose SAM, which derives frequency-channel (f−c) attention and frequency-time (f−t) attention simultaneously. The frequency-channel attention block computes the f−c attention plane, formulated as:

(1) $$X_{fc}^{\prime }\;=\;\sigma ({conv}_{2}\left(ReLU\;\left(BN\left({conv}_{1}\left(X_{fc})\right)\right)\right)\right),$$

where $X_{fc}\in R^{F\times C}$ denotes the cross-temporal pooled frequency-channel plane. Following [2], standard deviation pooling is introduced. ${conv}_{1}$ and ${conv}_{2}$ calculate attention weights on the plane. The kernel size of both conv1 and conv2 is 3 × 3. BN is a 2D batch normalization. The plane $X_{fc}^{\prime }$ is element-wise multiplied with input X for f−c wise attention. The f−t block calculates f−t wise attention in a similar manner, formulated as:

(2) $$X_{ft}^{\prime }\;=\;\sigma ({conv}_{2}\left(ReLU\;\left(BN\left({conv}_{1}\left(X_{ft}\right)\right)\right)\right)).$$

**Figure 1:**
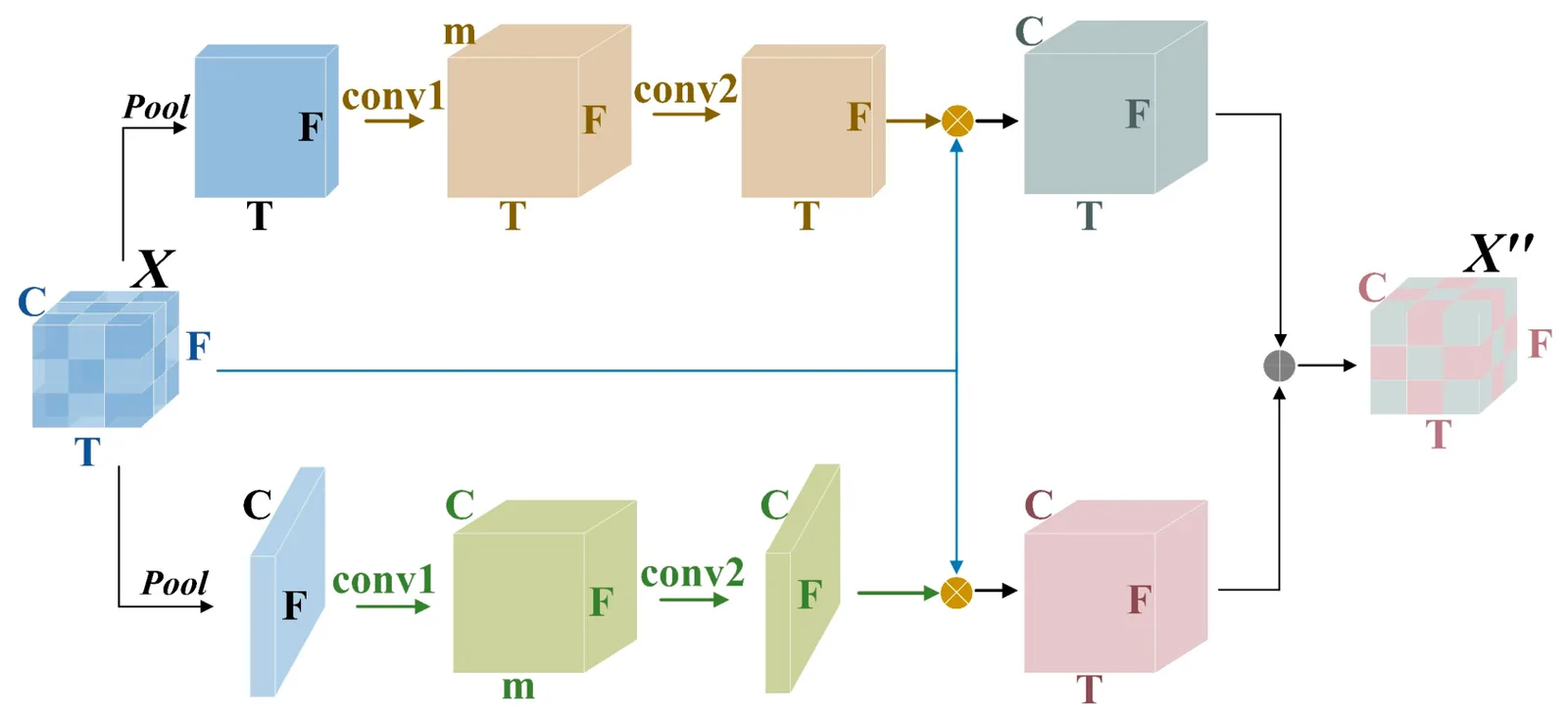
The architecture of the proposed SAM. It consists of an f-t block and an f-c block.

The f−c block and f−t block are applied in parallel, then f−c wise attention and f−t wise attention are averaged. SAM is illustrated in Figure 1. Parameters of SAM are derived from four convolutional operations. Each convolution kernel size is k×k, and the intermediate layer size is m. Thus, each convolutional layer has $k^{2}m$ parameters, and the attention module has $4k^{2}m$ parameters. In fact, the k and m are set to 3 and 8, with a total of 288 parameters, which is much lower than the parameters of CBAM and SE.

#### 2.1.2. Residual Network Architecture

The **SARN** architecture, which employs acoustic feature log-Mel filter-bank coefficients (Fbanks) as input, is composed of a backbone and two sub-networks, as shown in Table 1. The backbone is a streamlined ResNet-50, developed from [46], with an input size of 1×40×200 (channel×frequency×time). It is made up of 4 Residual Blocks with SAM (RB-SAM). SAM is located at the bottom of the residual block but before the residual connection. The speaker classification sub-network (Subnet 1) reshapes frequency and time dimensions into 512-dimensional (512 d) feature vectors using Global Average Pooling (GAP), similar to SE operations [36]. The speaker embedding sub-network (Subnet 2) flattens the dimensions of frequency and channel, and utilizes Attention Statistics Pooling (ASP) [36] for frame-level feature aggregation, then reduces the utterance-level features into 512 d by a FC layer (embedding layer). Although SARN incorporates two subnets, it does not impose a significant parameter burden compared to raw ResNet-50, with 10.6 M versus 25.6 M.

### 2.2. Transformer Approach

**Figure 2:**
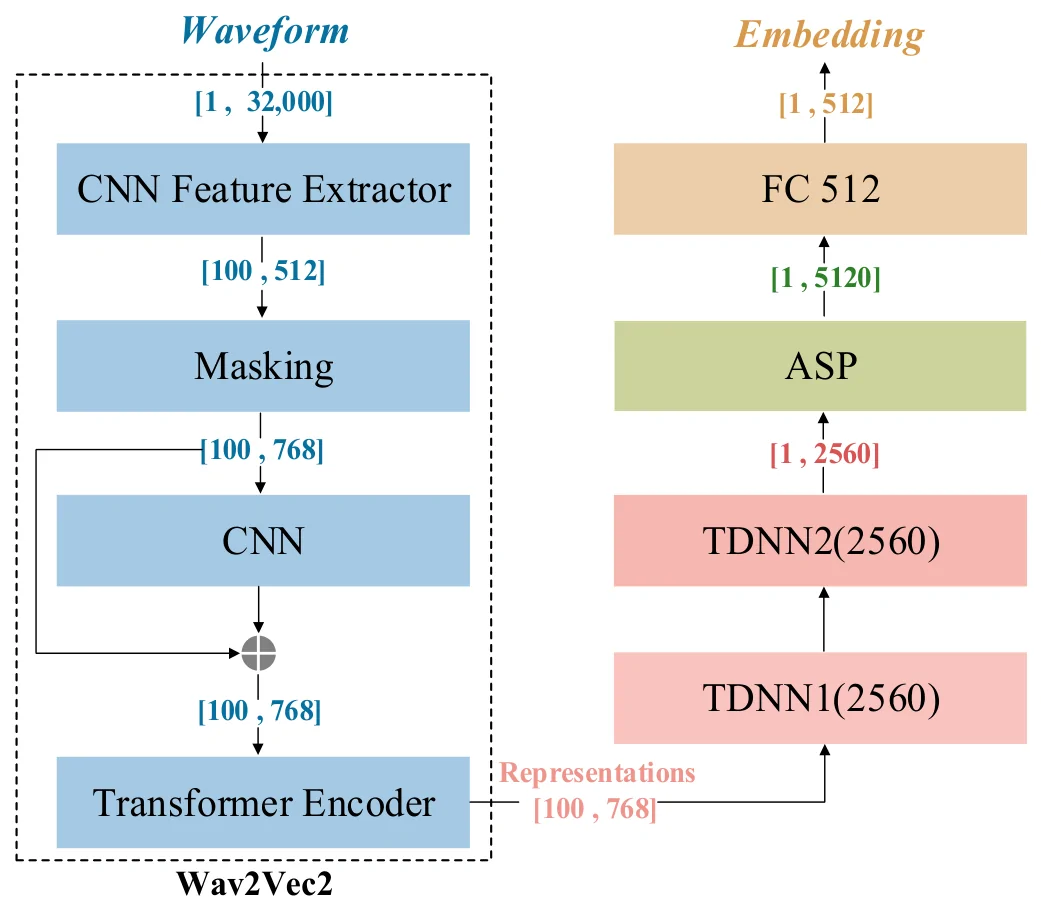
The architecture of **W2V2-TDNN**.

The Wav2Vec2 framework [33] is a renowned transformer-based tool for articulatory representation, pre-trained on large-scale, diverse and unlabeled data. It generates self-supervised representations from raw speech signals, widely utilized in speech recognition tasks (i.e., speech-to-text) due to its effectiveness in capturing speaker-specific characteristics, even under noisy and variable conditions. This paper proposes a Wav2Vec2-based TDNN (**W2V2-TDNN**) architecture designed for DSE extraction, as illustrated in Figure 2. **W2V2-TDNN** learns DSE descriptors from raw waveforms, eliminating the need for manual feature extraction. Its strong robustness to noise and reverberation enhances its applicability in real-world scenarios where background noise and fluctuating acoustics are prevalent.

#### 2.2.1. Wav2Vec2 Framework

The Wav2Vec2 framework processes waveforms through several steps, i.e., feature extractor, masking and transformer encoder [33]. The feature extractor consists of 7 consecutive 1D convolutions with 512 channels, each with a kernel size of (10, 3, 3, 3, 3, 2, 2) and a stride of (5, 2, 2, 2, 2, 2, 2). The first convolutional layer output is group normalized, ensuring that each of the 512-channel sequences has a zero mean and unit variance before the GELU activation is applied. Outputs of the remaining convolutional layers are activated using GELU, without any normalization operation. A single and shared FC layer projects fixed-size feature extractor outputs from 512 d to 768 d. Masking is applied over the whole sequence after projection. In order to obtain the relative positional embedding for the marked projected sequence, the masked sequence is convolved with a single layer, which has a kernel size of 128, a stride of 1, padding of 64, and 16 groups, followed by GELU activation. The positional embedding and marked sequence are summed and fed into the encoder with 12 consecutive transformer layers. Each transformer layer includes a residual multi-headed self-attention (MHSA) module and a residual 2-layer feed-forward network, consisting of 3072 and 768 units, respectively. The Wav2Vec2 representations are extracted at the encoder with a size of seq_len×768, where seq_len is determined by the input waveform length.

#### 2.2.2. Wav2Vec2-TDNN Architecture

Wav2Vec2 framework takes a 2 s waveform with size 1×32000 as input and applies MHSA mechanism in the deep layers. Inspired by [47], passing the Wav2Vec2 representations with size 100×768 to a statistical pooling layer directly can yield DSE descriptors of articulatory features. To further refine Wav2Vec2 time-series representations and emphasize speaker-specific information, a 2-layer TDNN block, an ASP layer, and a FC layer are incorporated following the Wav2Vec2 framework. The 2-layer TDNN block utilizes context 1 of the Wav2Vec2 representations and adjusts the representations on the time axis for 2560 d time-series representations. ASP computes mean and variance vectors with respect to time of the time-series representations. FC layer transforms the vectors into a fixed-size DSE descriptor of 512 d. Similar to SARN, an AAM-Softmax-based linear classification layer is adopted to fine-tune W2V2-TDNN for the downstream TI-SV task.

### 2.3. Hybrid Network Approach

In order to facilitate information sharing between acoustic features and articulatory representation at the utterance level, a hybrid network architecture is proposed to maximize the speaker information for performance boost. The architecture is named HSW-T, which stands for Hybrid network of SARN and W2V2-TDNN. HSW-T processes spectrogram-based information and self-supervised Wav2Vec2 representations individually at the frame level, and generates utterance-level DSE descriptors, which are aware of speaker traits present in two kinds of features.

#### 2.3.1. Hybrid Network Architecture

**Figure 3:**
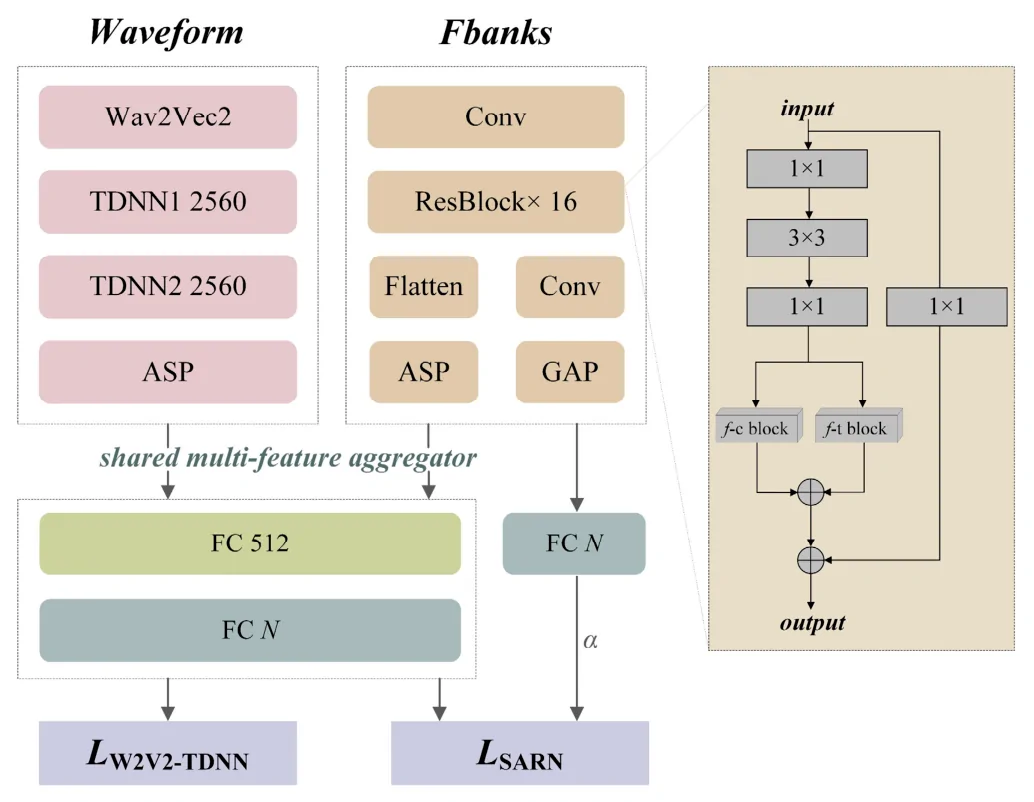
The architecture of **HSW-T**.

Building on the rapid success of the widely recognized TDNN-based x-vector [25], the HSW-T architecture is designed to capture frame-level features along the time axis and derive utterance-level DSE through frame-level feature aggregation. HSW-T is illustrated in Figure 3. Frame-level encoders of SARN and W2V2-TDNN are dedicated to processing each kind of input individually. SARN performs convolutional operations in the form of residual blocks to capture temporal context information from acoustic feature Fbanks, while W2V2-TDNN transforms the waveform into latent speech representations over a given context. Each ASP layer of two frame-level encoders computes statistics to get the gross utterance-level features. Compared to the independent utterance-level structures of SARN and W2V2-TDNN, the utterance-level multi-feature aggregator of HSW-T is shared between two frame-level encoders to extract DSE descriptors for speaker traits. The first FC layer has 512 units, and the second FC layer has N units, where N denotes the number of speaker classifications. The activations of the first FC layer (embedding layer) give deep embedding descriptors, as formulated:

(3) $$E_{t}=Wx_{t}+b,$$

where W and b are weight and bias vectors of the shared multi-feature aggregator, $x_{t}$ denotes 5120 d statistical vectors and $E_{t}$ denotes 512 d DSE descriptors. t represents vector type, and t∈{Fbanks, wav2vec2 representations}. While inferring, **HSW-T** is maneuverable to probe intrinsic information of speakers from any of the two frame-level features because of the shared utterance-level aggregator.

#### 2.3.2. Loss Function

Softmax loss function. It is extensively utilized in classification tasks, which can be explored to direct SARN for speaker classification; it is as follows:

(4) $$L_{Softmax}=-\frac{1}{N}\sum_{i=1}^{N}log\frac{e^{W_{y_{i}}^{T}x_{i}+b_{y_{i}}}}{\sum_{j=1}^{n}e^{W_{j}^{T}x_{i}+b_{j}}},$$

In this context, N represents the batch size, which is the number of samples processed before the model’s internal parameters are updated. The variable $x_{i}$ denotes the i-th input in the batch, while $y_{i}$ represents the corresponding true label for that input. The matrices W (weights) and vectors b (biases) are the trainable parameters of the model. According to a related study [48], the Softmax loss function does not explicitly promote intra-class compactness. This implies that DNNs trained using Softmax loss might struggle with generalization when it comes to DSE learning, which can negatively impact the model’s ability to generalize to new, unseen data.

AAM-Softmax loss function. It is an advanced derivative of the Softmax loss that applies a fixed margin between different classes. It is explored to direct SARN, W2V2-TDNN, as well as HSW-T for DSE learning. It is given by:

(5) $$L_{AAM-Softmax}=-\frac{1}{N}\sum_{i=1}^{N}log\frac{e^{s\left(\cos\left(\theta_{y_{i},i}+m\right)\right)}}{e^{s\left(\cos\left(\theta_{y_{i},i}+m\right)\right)}+\sum_{j=1,j\neq i}^{n}e^{scos\theta_{j,i}}\;},$$

where *s* is the scaling factor used to make sure the gradient is not too small, and *m* is the angular margin that enforces a stricter separation between classes. $\theta_{j,i}=arccos(\omega^{T}x)$ refers to the angular separation between i-th input and j-th column vector of the weight matrix. We set s=32 and m=0.1.

Complementary loss function. We integrate Softmax loss and AAM-Softmax loss, resulting in a complementary assembling loss function. Inspired by [49,50], a network comprising multiple subnets is designed to utilize a unified utterance-level structure, with consistent loss weights allocated across all subnets. Therefore, the loss of HSW-T is formulated as follows:

(6) $$L_{HSW-T}=L_{SARN}+L_{W2V2-TDNN},$$

and

(7) $$L_{SARN}=L_{AAM-Softmax}+{\alpha L}_{Softmax},$$

where $L_{SARN}$ is a trade-off between the Softmax of the classification subnet and the AAM Softmax of the embedding subnet, adapted by a hyperparameter α.

## 3. Experiment

### 3.1. Dataset

**VoxCeleb**. The well-known and open-source VoxCeleb1 [20] and VoxCeleb2 [22] are utilized for training and evaluating our proposed approach. The two datasets consist of videos of celebrities taken from YouTube. There is no speaker overlap between VoxCeleb1 and VoxCeleb2. In these two databases, each speaker has multiple recordings, and each recording has multiple speech utterances. For development, we adopt the dev set of Voxceleb2 as training data, which has a total of over 2300 h of data, with 5994 speakers and over 1 million speech utterances. For evaluation, we use the cleaned original test set (Vox1-O, 40 speakers, 37k trial pairs), extended test set (Voxl-E, 1251 speakers, 580k trial pairs) and hard test set (Vox1-H, 1190 speakers, 550k trial pairs) of Voxceleb1 as evaluation data. Compared to Vox-O, Vox-E and Vox-H have more test pairs, so they are considered more challenging.

**Aishell1** [21]. It is a mid-sized, open-source Mandarin Chinese dataset published by AISHELL Ltd. The dataset comprises over 140,000 utterances from 400 speakers. The recordings are collected using various devices, including microphones, Android phones, and Apple iPhones. All audio data in the AISHELL-1 dataset is stored as 16-bit streams with a single channel. The dataset is divided into three subsets: train, dev, and test. The train and dev subsets together include data from 380 speakers, encompassing over 130,000 utterances, while the test contains data from the remaining 20 speakers, comprising 7176 utterances. In this study, the train and dev sets are mixed for fine-tuning the target models to optimize the cross-domain model performance.

**FMAudio-v2**. This is a small-scale, self-collected Mandarin Chinese dataset containing approximately 600 utterances from 20 speakers. It is sourced from FM broadcasts and subsequently processed to convert the audio into 16-bit streams with a sampling rate of 16 kHz and a single channel. This study merges a test subset of Aishell1 and FMAudio-v2 as a mixture dataset named Aishell-FM to evaluate cross-domain performance of our proposed approaches. Aishell-FM includes microphone, Android phone, Apple iPhone and FM broadcasting channels. Aishell-FM simulates two cross-domain problems, namely cross-language (English to Chinese) and cross-channel (YouTube video to the above 4 channels).

### 3.2. Input Signal

**Log-Mel filter-bank coefficients (Fbanks)**. Typically, the Fbanks are prominent in TI-SV and serve as input for the SARN. 2 s fixed-length speech segments are chosen and converted to 1-channel, 16-bit streams with a 16 kHz sampling rate to ensure consistency, then converted using a sliding window width of 25 ms, window shift of 10 ms, FFT size of 512 and Mel filter bank of 40 for feature extraction. The process leads to a set of Fbanks with size 40×200 for any 2 s of speech. In order to improve the DSE robustness, data augmentation technologies are explored. Following [51], we augment the Fbanks on-the-fly with MUSAN noise and audio reverberation.

**Waveform matrix**. W2V2-TDNN requires input audio samples as raw waveform data. This data consists of floating-point numbers representing the amplitudes of the audio signals. It supports fixed sample rates of 16 kHz or 48 kHz and can handle durations of several seconds. For example, a 2 s speech segment sampled at 16 kHz forms a matrix with size 1×32000, whereas the same duration sampled at 48 kHz results in a matrix of size 1×96000. In this paper, waveform matrices with size 1×32000 of 2 s speech segments are adopted as W2V2-TDNN input.

### 3.3. Experimental Setup

In the paper, we utilize a Linux platform equipped with an Intel Core i9-9900K CPU running at 3.60 GHz, 64 GB of RAM, and an NVIDIA GeForce RTX 2080 Ti GPU. The software configuration includes Python 3.5 and CUDA 11.1, and the proposed networks are realized using the PyTorch 1.4 toolkit. Adam optimizer with weight decay of $2\times 10^{-5}$ is utilized in this study. The learning rate, weight decay, and batch size were selected based on a grid search on the validation set. The initial learning rate is set to 0.1. It is decreased by a factor of 10 after the loss function value fails to decrease significantly for two epochs. The reduction continues until the learning rate reaches $1\times 10^{-8}$ or after 40 epochs, whichever occurs first. Chunks of speech utterances are randomly selected, which gives a batch of Fbanks with size $R^{B\times 1\times 40\times 200}$ and a batch of raw waveform matrix $R^{B\times 1\times 32000}$, where the batch size B is set to 64 during training, and set to 1 during evaluation. The Wav2Vec2 framework is frozen during training to preserve its pre-trained representations. Two types of 512 d DSE can be extracted at corresponding embedding layers. Equal Error Rate (EER), where the false acceptance rate equals the false rejection rate, ref. [20] is employed for evaluating in forthcoming experiments. Cosine distance [52] is used to quantify the similarity between a pair of DSE descriptors of the same type, as formulated:

(8) $$Score\left(E_{t,1},E_{t,2}\right)=\frac{E_{t,1}^{T}E_{t,2}}{\left\|E_{t,1}\right\|\left\|E_{t,2}\right\|},$$

where t represents DSE type, and t∈{Fbanks,wav2vec2 representations}.

## 4. Results

### 4.1. Results of Hyper-Parameter α on Vox-O

**Figure 4:**
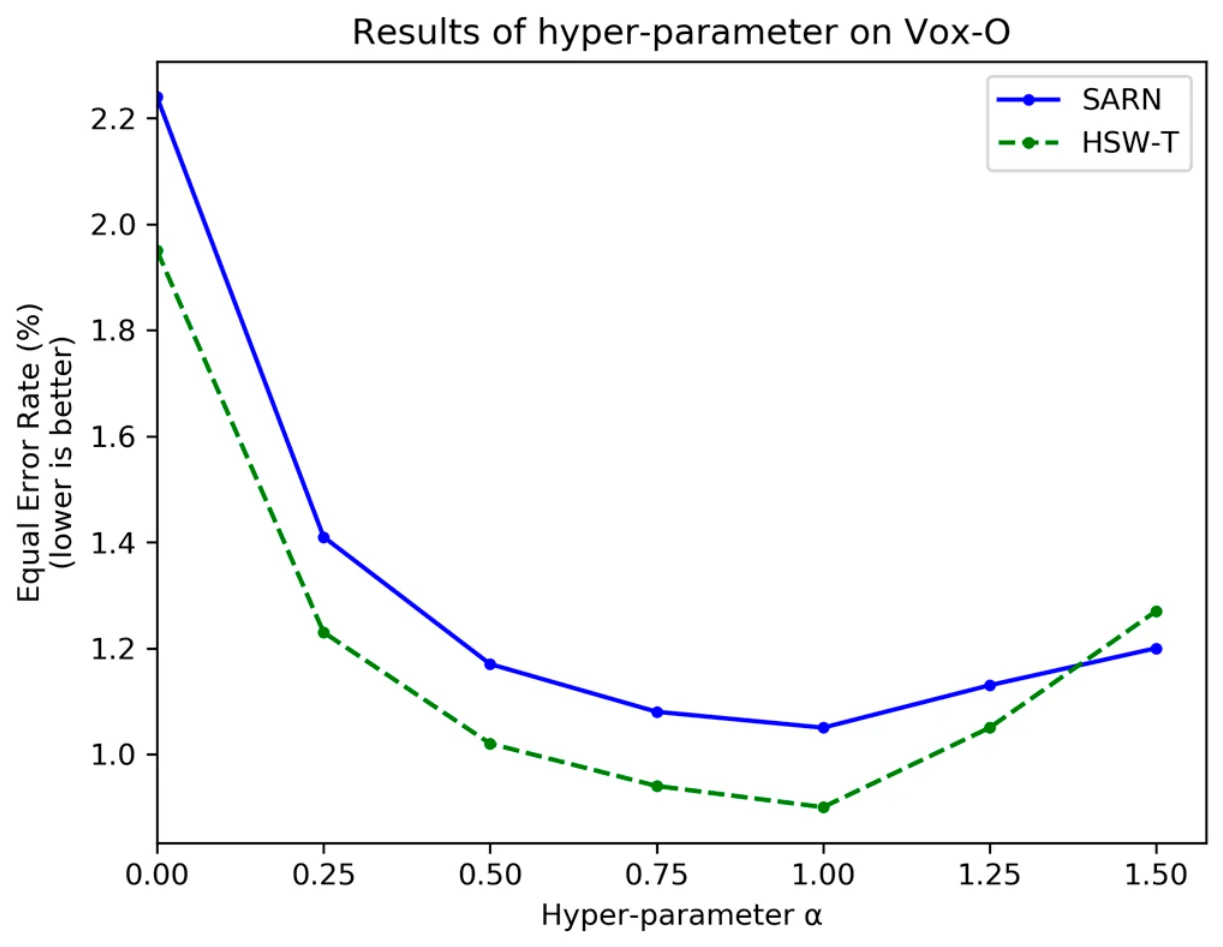
Results of **SARN** and **HSW-T** under different hyper-parameters.

Figure 4 shows the experimental results of SARN and HSW-T on Vox-O under different hyper-parameters. Optimal α value in Equation (6) is explored in these experiments. In the figure, the best EER result of SARN is 1.05%, while the best EER result of HSW-T is 0.9%. Both of these best EER results are achieved with α=1. The results mean a 1:1 combination of AAM-Softmax loss and Softmax loss is the best setting. Increasing or decreasing this value leads to an increase in EER. Therefore, we set α=1 in the following experiments.

### 4.2. Ablation Experiment Results on Vox-O

Table 2 shows the ablation results of SARN by controlling the variable. CBAM and SE are both plug-and-play attention modules, serving as baselines for comparison with the proposed SAM. The table shows a marked performance degradation when the attention modules are removed, while all other conditions remain unchanged (Residual Network without attention modules, RN), proving the efficacy of the three attention mechanisms throughout the networks. Notably, the performance advantage of SARN is witnessed, achieving the best EER result of 1.05% and the lowest learnable parameters of 10.59 M, compared to the CBAM-inserted Residual Network (CBRN) and the SE-inserted Residual Network (SERN).

Table 3 presents the ablation results of W2V2-TDNN, compared with the W2V2-TDNN architecture without a 2-layer TDNN block (W2V2). In fact, passing the Wav2Vec2 representations directly to the pooling layer can also lead to model convergence. However, when the Wav2Vec2 representations are first passed through the TDNN layers before the pooling layer, the experimental result shows a significant improvement, with the EER decreasing from 1.30% to 1.01%. This indicates that the TDNN block functions to extract time-series representations and emphasize speaker-specific information, which aids in learning discriminative DSE descriptors and contributes to performance enhancement.

Table 4 shows the ablation results of networks with independent utterance-level structures. Every network is stand-alone and followed by its proprietary utterance-level structure; that is to say, the utterance-level structures are unshared. Similarity score of Fbanks embedding descriptors ${(s}_{1})$ and the similarity score of Wav2Vec2 representation embedding descriptors ${(s}_{2})$ are used to investigate the impact of speaker-specific information in two features on speaker recognition. The fusion of these similarity scores ${(s}_{1}+s_{2})$ aims to explore the potential of information sharing to enhance TI-SV performance. $s_{1}$ and $s_{2}$ are calculated as Equation (7). The speaker-specific information in Wav2Vec2 representations is more abundant compared to Fbanks, with an EER of 1.01% vs. 1.05%. It demonstrates the potential of deep learning-based speech representations over traditional hand-crafted features in TI-SV. Furthermore, the fusion ${(s}_{1}+s_{2})$ results in a performance boost, achieving an EER of 0.96%, indicating that these two features provide complementary information that enhances performance.

Table 5 shows the ablation results of networks with shared utterance-level structures (multi-feature aggregator). The results show a significant performance improvement compared to networks with independent utterance-level structures. Strengthened by shared aggregators, the enhanced **SARN** (**eSARN**) and the enhanced **W2V2-TDNN** (**eW2V2-TDNN**) achieve 6.67% and 8.91% improvements for stand-alone **SARN** and **W2V2-TDNN** in Table 4. The fusion ${(S}_{1}+S_{2})$ of **HSW-T** achieves a similar performance boost compared to the fusion ${(s}_{1}+s_{2})$ of two individual networks, and it registers an improvement of 6.25%. $S_{1}$ and $S_{2}$ are calculated as Equation (7). The disparity in performance between fusion ${(S}_{1}+S_{2})$ and fusion ${(s}_{1}+s_{2})$ can be attributed to the shared multi-feature aggregator: **HSW-T** is an interconnected hybrid network, and its two subnets are optimized simultaneously, which makes it more generalized by exploring related information from two directions.

### 4.3. Comparison of the Proposed Approach with Existing Benchmarks on Vox-O, Vox-E and Vox-H

Our proposed approaches are compared with several recent works. The experimental results are reported on EER and minDCF in Table 6. In the development stage, all models are developed on the dev set of Voxceleb2. The Wav2Vec2 parts are frozen while tuning for TI-SV. In the evaluation stage, these models are evaluated on the Vox-O, Vox-E and Vox-H sets, respectively. In the table, SARN, W2V2-TDNN, HSW-T, ResNet-34 with SE [2], ResNet-34 with fw-SE [2], as well as ECAPA-TDNN with SE [36] are all significantly superior to ResNet-18 [31,36], ResNet-34 [31], and E-TDNN [36] without an attention module, proving these intermediate plug-and-play attention modules are helpful for TI-SV performance improvement. SARN outperforms most previous approaches, reflecting that the two-branch architecture, representing two tasks, is complementary to enhance the DSE discrimination. W2V2-TDNN is superior to SARN. It conveys that the speaker-related information of deep learning-based Wav2Vec2 representations is richer than hand-crafted features, proving that Wav2Vec2 representations are conducive to learning more discriminative DSE. Using shared utterance-level structure for multi-feature aggregation, the hybrid HSW-T achieves a further performance boost, demonstrating the best performance on the minDCF metric and relatively superior performance on the EER metric. It is worth noting that the 2.02% EER on Vox-H corresponds to ResNet-34 with fw-SE, which slightly outperforms HSW-T (2.04%) in this specific set.

### 4.4. Cross-Domain Experimental Results on Aishell-FM

The cross-domain performance comparisons of our proposed approaches are shown in Table 7. We conduct experiments on SARN, W2V2-TDNN and HSW-T, respectively. Following the original Wav2Vec2 paper [38] and inspired by [53], we fine-tune SARN with resident blocks frozen, W2V2-TDNN with Wav2Vec2 frozen, as well as HSW-T with both resident blocks and Wav2Vec2 frozen. Under the condition of cross-domain, HSW-T achieves the best performance with an EER of 1.08%, which is close to the domain-matched condition with an EER result of 0.9%, and achieves an inference latency of approximately 90 ms per utterance, meeting the typical sub-100 ms requirement for real-time access control applications. Notably, SARN is significantly superior to W2V2-TDNN with an EER of 1.22% vs. 1.96%, which is exactly opposite to the situation of domain-matched. Compared with the result gap of W2V2-TDNN between domain-matched and domain-mismatched, the result gap of SARN is smaller. In the aspect of the attention mechanism, SAM can be regarded as a 3D (time, frequency and channel) domain adapter, which extracts knowledge from each domain and fuses it with speaker-related information, effectively mitigating domain-related disparities, suggesting that 3D SAM has potential in cross-domain scenarios. The large gap for W2V2-TDNN is attributed to the English vs. Mandarin domain mismatch, as the frozen Wav2Vec2 cannot adapt to Mandarin acoustic characteristics.

## 5. Conclusions

In this paper, we propose a hybrid method for TI-SV, designed to address both short-segment speech and cross-domain scenarios. The hybrid method integrates the robust capabilities of ResNet and Wav2Vec2 to enhance performance, particularly for access control applications. First, a CNN method employing a modified ResNet-50 with stereoscopic attention modules is introduced to effectively capture speaker-related information from acoustic features. The stereoscopic attention module models frequency-channel and frequency-time interactions to compute attention weights within a three-dimensional feature space, all while maintaining a low parameter count. Second, a transformer method is presented, combining Wav2Vec2 and TDNN architectures to extract distinctive articulatory patterns directly from raw signals. This method exhibits strong robustness to noise and reverberation, making it highly effective in real-world environments with background noise. To unify the strengths of both approaches, a multi-feature aggregator is proposed, facilitating utterance-level information sharing and enabling the construction of a unified framework. Experimental evaluations on the VoxCeleb validate the effectiveness of both CNN and transformer methods and demonstrate that the hybrid method significantly outperforms both. The hybrid method also exhibits superior performance compared to several state-of-the-art methods and demonstrates its robustness in cross-domain conditions when tested on a mixed dataset. Future work will examine the comparative effects of freezing versus fine-tuning the Wav2Vec2 backbone on cross-domain performance.

## Figures and Tables

**Table 1:** The SARN Architecture. N denotes the number of speakers.

Type	Operation	Size
Backbone		
input	-	1×40×200
conv	con2d, 7×7, 16	16×40×200
RB-SAM-1	$\left[\begin{array}{c} 1\times 1,16,(1,\;1)\\ 3\times 3,\;16,(1,\;1)\\ \begin{array}{c} 1\times 1,\;64,(1,\;1)\\ SAM \end{array} \end{array}\right]$ × 3 ^1^	64×40×200
RB-SAM-2	$\left[\begin{array}{c} 1\times 1,\;32,\;(2,\;2)\\ 3\times 3,\;32,(2,\;2)\\ \begin{array}{c} 1\times 1,\;128,(2,\;2)\\ SAM \end{array} \end{array}\right]$ × 4 ^1^	128×20×100
RB-SAM-3	$\left[\begin{array}{c} 1\times 1,\;64,\;(2,\;2)\\ 3\times 3,\;64,(2,\;2)\\ \begin{array}{c} 1\times 1,\;256,(2,\;2)\\ SAM \end{array} \end{array}\right]$ × 6 ^1^	256×10×50
RB-SAM-4	$\left[\begin{array}{c} 1\times 1,\;128,\;(2,\;2)\\ 3\times 3,\;128,(2,\;2)\\ \begin{array}{c} 1\times 1,\;512,(2,\;2)\\ SAM \end{array} \end{array}\right]$ × 3 ^1^	512×5×25
Speaker classification sub-network (Subnet 1)		
pool	GAP, 5×25	512×1
FC	FC N	N×1
loss	Softmax	-
Speaker embedding sub-network (Subnet 2)		
flatten	-	2560×25
ASP	-	5120×1
FC (embedding)	FC 512	512×1
FC	FC N	N×1
loss	AAM−Softmax	-

^1^ Each block is denoted as (kernel size, output channels, stride).

**Table 2:** Ablation study results of SARN.

	Network	Attention	Params	EER
1	RN	No	10.59 M	1.34%
2	CBRN	CBAM	10.74 M	1.22%
3	SERN	SE	10.74 M	1.16%
4	**SARN**	SAM	10.59 M	**1.05%**

**Table 3:** Ablation study results of **W2V2-TDNN**.

	Network	TDNN	Pooling	EER
1	W2V2	No	ASP	1.30%
2	**W2V2-TDNN**	2-layer	ASP	**1.01%**

**Table 4:** Networks with independent utterance-level structures results.

	Network	Embedding of	Score	EER
1	**SARN**	Fbanks	$S_{1}$	1.05%
2	**W2V2-TDNN**	Wav2Vec2 representations	$S_{2}$	1.01%
3	**SARN&W2V2-TDNN**	Fbanks & Wav2Vec2 representations	$S_{1}$ + $S_{2}$	**0.96%**

**Table 5:** Networks with shared utterance-level structure results.

	Network	Embedding of	Score	EER
1	**eSARN**	Fbanks	$S_{1}$	0.98%
2	**eW2V2-TDNN**	Wav2Vec2 representations	$S_{2}$	0.92%
3	**HSW-T**	Fbanks & Wav2Vec2 representations	$S_{1}+S_{2}$	**0.90%**

**Table 6:** The results of different models. In our work, P_(target) = 0.01, C_(miss) = C_(fa) = 1 are adopted to compute the minDCF.

	Backbone	Attention	Vox-O		Vox-E		Vox-H	
			EER	minDCF	EER	EER	minDCF	EER
Yadav et al. [31]	PResNet-50v2	CBAM	2.52%	-	-	-	-	-
Yadav et al. [31]	PResNet-50v2	*f*-CBAM	2.46%	-	-	-	-	-
Li et al. [2]	ResNet-34	SE	0.96%	-	1.20%	-	2.20%	-
Li et al. [2]	ResNet-34	fw-SE	0.96%	-	1.12%	-	2.02%	-
Choi et al. [39]	ResNet-18	-	2.03%	0.1410	2.08%	0.1410	3.73%	0.2300
Choi et al. [39]	ResNet-34	-	1.60%	0.1080	1.73%	0.1190	3.20%	0.1940
Desplanques et al. [32]	E-TDNN	-	1.26%	0.1399	1.37%	0.1487	2.35%	0.2153
Desplanques et al. [32]	ResNet-18	-	1.47%	0.1772	1.60%	0.1789	2.88%	0.2672
Desplanques et al. [32]	ECAPA-TDNN	SE	1.01%	0.1274	1.24%	0.1418	2.32%	0.2181
**SARN**	modified ResNet-50	SAM	1.05%	0.0923	1.25%	0.1112	2.50%	0.2034
**W2V2-TDNN**	Wav2vec2	MHSA	1.01%	0.0930	1.18%	0.1261	2.15%	0.2059
**HSW-T**	hybrid network	SAM+MHSA	**0.90%**	**0.0912**	**1.05%**	**0.1103**	2.04%	**0.1906**

**Table 7:** Cross-domain experimental results.

	Network	Test Set	Cross-Domain	EER
1	**SARN**	Vox-O	No	1.05%
2	**W2V2-TDNN**	Vox-O	No	1.01%
3	**HSW-T**	Vox-O	No	0.9%
4	**SARN**	Aishell-FM	Yes	1.22%
5	**W2V2-TDNN**	Aishell-FM	Yes	1.96%
6	**HSW-T**	Aishell-FM	Yes	1.08%

## Data Availability

The original contributions presented in this study are included in the article. Further inquiries can be directed to the corresponding authors.

## Abbreviations

The following abbreviations are used in this manuscript:

SV	Speaker Verification
TD-SV	Text-Dependent Speaker Verification
TI-SV	Text-Independent Speaker Verification
GMM-UBM	Gaussian Mixture Model-Universal Background Model
DSE	Deep Speaker Embedding
CBAM	Convolutional Block Attention Module
SE	Squeeze-and-Excitation
SARN	Stereoscopic Attention Residual Network
W2V2-TDNN	Wav2Vec2-based TDNN
MFA	Multi-scale Frequency-channel Attention
SARN	Stereoscopic Attention ResNet
SAM	Stereoscopic Attention Module
MLP	Multi-Layer Perceptron
GAP	Global Average Pooling
ASP	Attention Statistics Pooling
HSW-T	Hybrid network of SARN and W2V2-TDNN
